# Editorial: Antimicrobial use and stewardship in pediatrics

**DOI:** 10.3389/fped.2023.1191650

**Published:** 2023-04-20

**Authors:** Zikria Saleem

**Affiliations:** Department of Pharmacy Practice, Faculty of Pharmacy, Bahauddin Zakariya University, Multan, Pakistan

**Keywords:** antimicrobial use, antimicrobial stewardship (AMS), pediatrics, antimicrobial resistance (AMR), policy

**Editorial on the Research Topic** Antimicrobial use and stewardship in pediatrics

Antimicrobial resistance (AMR) is a growing global public health threat that undermines the effectiveness of many life-saving medications. The overuse and misuse of antimicrobials in humans and animals are major drivers of AMR (1). Children are particularly vulnerable to AMR due to their developing immune systems, higher infection rates, and frequent exposure to antibiotics. In low and middle income countries (LMIC) like Pakistan, rate of antimicrobial use in pediatrics is very high (2). In this context, antimicrobial stewardship (AMS) in pediatrics is critical to optimize antimicrobial use and mitigate the risk of AMR (3). AMS is a comprehensive approach to promote the appropriate use of antimicrobials by optimizing selection, dose, duration, and route of administration. AMS programs involve a multidisciplinary team that includes clinicians, pharmacists, microbiologists, infection preventionists, and other healthcare providers (4).

Pediatric AMS is a challenging and complex area that requires tailored strategies and continuous monitoring. Children have unique pharmacokinetic and pharmacodynamic profiles that influence the efficacy, safety, and tolerability of antimicrobials. Children may also have limited therapeutic options due to age-specific indications, drug formulations, and regulatory restrictions. Furthermore, pediatricians may face pressure from parents, other healthcare providers, and societal expectations to prescribe antimicrobials, even when they are not indicated (3). To address these challenges, pediatric AMS programs need to be evidence-based, collaborative, and adaptable to local contexts. They should involve education and training for healthcare providers, patients, and families to raise awareness of the risks of AMR and the benefits of AMS. They should also incorporate clinical decision support tools, guidelines, and audits to guide antimicrobial prescribing practices and monitor their impact. AMS programs should leverage innovative technologies, such as rapid diagnostic tests and telemedicine, to improve diagnosis and treatment of infections (5).

Three articles are published in current issue. The first study found that severe community-acquired pneumonia is a leading cause of death in children under 5 years old, and that clinical characteristics, comorbidities, and medications are associated with mortality (Cao et al.). The second study found that a multifaceted antimicrobial stewardship program in primary care pediatricians resulted in a significant reduction in overall antibiotic prescription and the use of broad-spectrum antibiotics Pagano et al. The third study highlighted the potential of metagenomics by next-generation sequencing (mNGS) in improving the identification of pathogens in neonatal and pediatric sepsis, particularly in cases with negative cultures and unusual microorganisms, which could help to improve the rational use of antibiotics (Agudelo et al.).

In conclusion, pediatric AMS is a critical component of the global efforts to combat AMR and ensure the optimal use of antimicrobials in children. AMS programs in pediatrics require a multidisciplinary and collaborative approach that involves healthcare providers, patients, and families. Such programs should be evidence-based, tailored to local contexts, and continuously monitored to ensure their effectiveness and sustainability. The time to act is now, and we call on all stakeholders to join forces to promote pediatric AMS and safeguard the health of our children and future generations.
